# Sickle Cell Disease in Pregnancy: Trend and Pregnancy Outcomes at a Tertiary Hospital in Tanzania

**DOI:** 10.1371/journal.pone.0056541

**Published:** 2013-02-13

**Authors:** Projestine S. Muganyizi, Hussein Kidanto

**Affiliations:** Department of Obstetrics & Gynecology, School of Medicine, Muhimbili University of Health and Allied Sciences (MUHAS), Dar es Salaam, Tanzania; Instituto de Ciencia de Materiales de Madrid - Instituto de Biomedicina de Valencia, Spain

## Abstract

SCD in pregnancy is associated with increased adverse fetal and maternal outcomes. In Tanzania where the frequency of sickle cell trait is 13% there has been scanty data on SCD in pregnancy. With progressive improvement in childhood survival the burden of SCD in pregnancy will increase. We analyzed all deliveries at Muhimbili National Hospital (MNH) from 1999 to 2011. Fetal and maternal outcomes of SCD deliveries were compared with non-SCD. Data were analyzed using IBM SPSS statistics version 19. Chi square and Fisher Exact tests were used to compare proportions and the independent t-test for continuous data. To predict risks of adverse effects, odds ratios were determined using multivariate logistic regression. A p-value<0.05 was considered significant. In total, 157,473 deliveries occurred at MNH during the study period, of which 149 were SCD (incidence of 95 SCD per 100,000 deliveries). The incidence of SCD had increased from 76 per 100,000 deliveries in the 1999–2002 period to over 100 per 100, 000 deliveries in recent years. The mean maternal age at delivery was lower in SCD (24.0±5.5 years) than in non-SCD deliveries (26.2±6.0 years), p<0.001. Compared with non-SCD (2.9±0.7 Kg), SCD deliveries had less mean birth-weight (2.6±0.6 Kg), p<0.001. SCD were more likely than non-SCD to deliver low APGAR score at 5 minutes (34.5% *Vs* 15.0%, OR = 3.0, 95%CI: 2.1–4.2), stillbirths (25.7% *Vs* 7.5%, OR = 4.0, 95%CI: 2.8–5.8). There was excessive risk of maternal deaths in SCD compared to non-SCD (11.4% *Vs* 0.4%, OR = 29, 95%CI: 17.3–48.1). The leading cause of deaths in SCD was infections in wholly 82% in contrast to only 32% in non-SCD. In conclusion SCD in pregnancy is an emerging problem at MNH with increased adverse fetal outcomes and excessive maternal mortality mainly due to infections.

## Introduction

Sickle cell disease (SCD) is a group of inherited single-gene autosomal recessive disorders caused by the sickle cell gene which affects hemoglobin structure [1]–[6]. SCD encompasses Sickle cell Anemia (SCA) which possesses SS genotype, some heterozygous conditions of the S gene and other clinically abnormal hemoglobins such as beta thalassemia, hemoglobin C, D, E and others. The combination of Sickle gene with any of these abnormal hemoglobins will lead to a similar clinical picture of SCD although the severity of the disease and prevalence differ. SCD is believed to originate from Sub-Saharan Africa and Middle East, hence high prevalence is found among populations in these regions and their descendants elsewhere in the world [7]. It is estimated 300,000 children are born each year with SCD with 75% of them living in Sub-Saharan Africa [8]–[9]. SCD is a major public health problem in East Africa. In Uganda a mean frequency of 20% Sickle cell trait is documented, with an expected 25,000 babies born with SCD each year [10]. Similarly high annual incidence of SCD births can be expected in Tanzania where the quoted frequency of Sickle cell trait is 13% [11].

SCD is associated with increased childhood morbidity and mortality. World Health Organization estimates 50–80% SCD patients in Sub-Saharan Africa will die before adulthood [9]. In Tanzania the most vulnerable children to SCD mortality are those under the age of five [11]. Most of SCD deaths in childhood could be prevented by routine newborn screening and targeted standard interventions. In countries where effective measures are undertaken to prevent morbidity and mortality due to SCD, 94–99% of the children survive to age 18–20 years and life expectancy is on average at least mid-50s [4]–[5].

Patients with SCD who survive childhood and who become pregnant are likely to suffer aggravated morbidity and mortality. International evidence on pregnancy outcome among SCD has been inconsistent. Low and middle income countries generally report increased maternal and perinatal morbidity and mortality in association with SCD [12]–[15]. In Jamaica where 10% of the population has the S gene, the risk of maternal deaths is 7–11 times higher in SCD comparing to the general population [12]. Studies in high income countries generally report more favorable fetal outcomes without appreciable risk for increased maternal mortality [15]–[17]. Although there is scanty literature on SCD in pregnancy from Sub-Saharan Africa, the little available studies suggest poor pregnancy outcomes in SCD with maternal mortality varying from 1.8% to 9% [18]–[19]

In Tanzania pregnancy outcomes of SCD have not been systematically documented. Generally the national maternal mortality ratio has slightly decreased in the last decade from 578 to the current 454/100,000 live births based on the 2010 Tanzania Demographic and Health Survey (TDHS) data. Likewise, National statistics have reported a decrease in under-five mortality from 106 deaths per 1000 live births 5–9 years ago down to the current 81 deaths per 1000 live births [20]. Since most of SCD deaths in Tanzania occur among children under the age of five years [11], improved general childhood survival can result in increased SCD population of women in the reproductive age and a rise in SCD deliveries. This study was conducted in order to provide baseline data on the trend of SCD deliveries over the past 13 years (1999–2011) and adverse maternal and fetal outcomes among SCD deliveries in Tanzania. The objective was to compare outcomes of SCD deliveries with that of the general population of women (non-SCD deliveries) who delivered at MNH in the same period. The results in this article have effectively filled the gap in knowledge about SCD in pregnancy in Tanzania.

## Methodology

### Study setting

Muhimbili National Hospital (MNH) is the largest consultant hospital in the United Republic of Tanzania being situated in Dar es Salaam, the country's largest city. According to the 2002 national population Census, the city has a total population of about 3.4 million with annual growth rate of 4.3%. The maternity unit is affiliated to the department of obstetrics and gynaecology in the MNH structure. The unit receives referred pregnant women from Dar es Salaam district hospitals as well as other hospitals from within the city. Occasionally it receives patients from other nearby regions. About 40 women deliver at this unit each day. The MNH also serves as teaching hospital for the Muhimbili University Health and Allied Sciences (MUHAS).

### The obstetric database

This study utilized information stored in the MNH electronic obstetric database from 1^st^ January, 1999 to 31^st^ December, 2011. The database was established in 1998 and since then data have been prospectively entered. Patients admitted to the MNH labor ward bring their antenatal (RCH4) cards and the information is entered into the admission book on arrival. Chronic maternal diseases such as SCD are indicated in this card. After delivery, antenatal information such as antenatal diagnoses and risk factors from the RCH4 card are computerized together with data on labor, maternal and neonatal outcomes from the midwifery book. The obstetric database thus contains information on: maternal age, marital status, parity, antenatal diagnoses, APGAR scores at one and five minutes, birth weight, maternal and fetal outcomes as well as maternal complications. MNH has a maternal death audit committee which discusses all maternal deaths regarding the causes and the information is entered in the database. Validity of data is ensured by a data quality program run weekly and the validity checks of the data done twice annually. The latter is done by annual comparison, between the information in the ledgers with the information, in the database for selected variables. From the electronic obstetric database we identified all deliveries during the entire study period and analyzed their data.

### Data processing and analysis

All the needed variables were scrutinized for missing or inappropriately entered data. It was agreed at the start of analysis that any variable with 5% or more missing data will be excluded. A comparison was made between SCD deliveries and the general population of deliveries without a diagnosis of SCD. In multiple pregnancies, only data for the first born were considered. Comparison of means for continuous variables was done using the independent t-test. Proportions were compared using Chi square or Fisher exact test as appropriate. In all the comparisons, p-value less than 0.05 were considered significant.

### Ethics statement

This study was ethically approved by the MUHAS ethical and publication committee and the permission to use the database was obtained from MNH Authorities.

## Results

During the study period there were 157,473 deliveries at MNH of which 151,518 were singleton. There were 149 SCD deliveries which makes the incidence of 95 SCD per 100,000 deliveries. Overall, the median age at delivery was 26(±6.0) years with a range of 12–50 years. From Table 1, most deliveries (84.9%) were to mothers in the relatively low risk age range of 18–34 years. Attendance to antenatal clinic was 99.5% of all deliveries. Around 18% of all the deliveries had low birth weight, and 7.1% were stillbirths. Overall there were 706 maternal deaths which is equivalent to 448 deaths per 100,000 deliveries at this hospital.

**Table 1:** Characteristics of all deliveries in MNH from 1999 to 2011.

Characteristics	Number*	Percentage
**Age at delivery (years)**		
<18	7139	4.6
18–34	130833	84.9
>34	16141	10.5
**Status of referral**		
Referred	28935	18.4
Not referred	128332	81.6
**Parity**		
0–5	149069	95.9
>5	6341	4.1
**Gestation Age(weeks)**		
<33	4247	4.0
33–36	28067	27.0
>36	71811	69.0
**HIV status** †		
Negative	33401	93.9
Positive	2187	6.1
**Antenatal visits**		
No visit	716	0.5
1–4	66140	42.5
5 or more	88550	57.0
**Mode of delivery**		
Vaginal	108068	70.4
Cesarean section	45439	29.6
**APGAR score at 5 minutes**		
<7	23102	15.0
7–10	130918	85.0
**Stilbirths**		
Yes	12231	7.1
No	141790	92.9
**Birth weight(Kg)**		
<2.5	27374	17.9
2.5 or above	125848	82.1
**Maternal outcome**		
Alive	154650	99.5
Died	706	0.45

* Missing data are not included. In all variables, missing data accounted for less than 5%.

† In only 35,588 (22.6%) of all deliveries was HIV status known.

**Figure 1:**
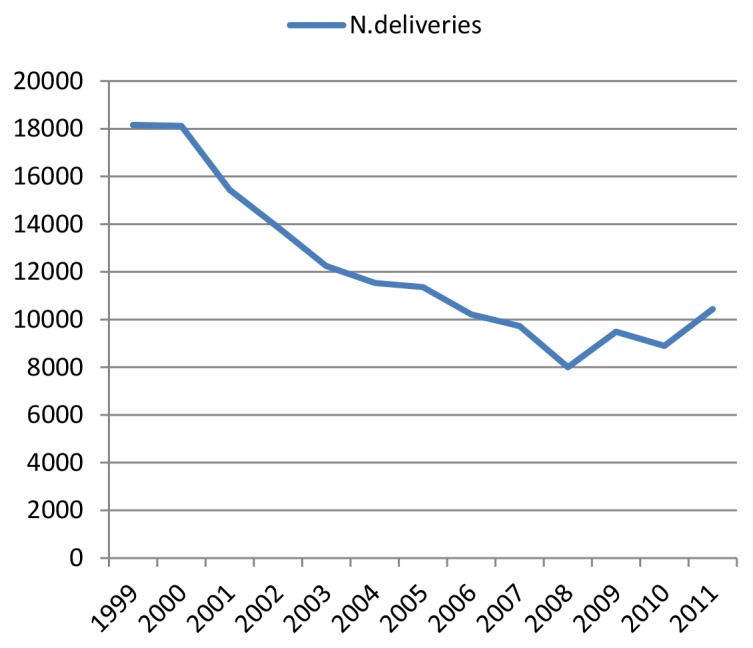
Trend of annual deliveries at MNH.

During the study period deliveries at MNH had gone down by over 50%. Figure 1 indicates a steady decline in all annual deliveries from 18,158 in 1999 to 8000 in 2008. Since 2009 there has been a gradual increase to 10441 deliveries in 2011.

**Figure 2:**
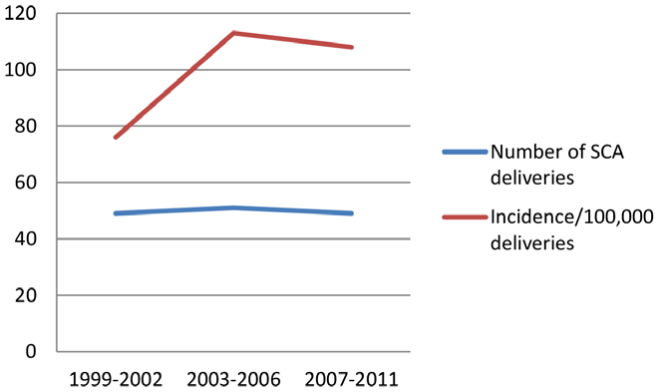
Trend of SCD deliveries at MNH.

The incidence of SCD deliveries had increased from 76 per 100,000 deliveries in the first four years (1999–2002) to over 100 per 100,000 deliveries in subsequent similar periods (Figure 2).

A comparison of characteristics of SCD deliveries and the general population (non-SCD deliveries) is demonstrated in Table 2. SCD deliveries were significantly younger and of lower parity than non-SCD deliveries. The mean number of antenatal visits, the gestation age at delivery and mode of delivery were not statistically different among the two groups.

**Table 2:** Comparison of prenatal characteristics and pregnancy outcomes among SCD and non SCD deliveries at MNH, 1999–2011.

Characteristics	SCD*	Non-SCD*	p-Value	OR	95%CI
**Mean Age at delivery (±SD yrs)**	24.0 (±5.5)	26.2(±6.0)	<0.001		
**Status of referral**					
Referred	107 (72.3)	126972 (81.9)			
Not referred	41 (27.7)	28115 (19.1)	0.003	1.7	1.2–2.5
**Mean Parity**	1.7(±1.0)	2.2(±1.5)	<0.001		
**Parity**					
0–5	148(99.3)	148921(95.9)			
>5	1 (0.7)	6340 (4.1)	0.01	0.2	0.08–0.7
**Mean Gestation Age(weeks)**	37.0(±2.3)	37.3(±2.1)	0.1		
**Premature delivery**					
Yes (<37)	41(36.9)	32273(31.0)	0.18	1.3	0.9–1.9
No (37 or more)	70(63.1)	71741(69.0)			
**Mean Antenatal visits**	5.3 (±2.4)	5.3(±2.6)	0.8		
**Mode of delivery**					
Vaginal	109(73.2)	107959(70.4)			
Cesarean section	40(26.8)	45399(29.6)	0.5	0.9	0.6–1.2
**APGAR score at 5 minutes**					
<7	51(34.5)	23051(15.0)			
7–10	97(65.5)	130821(85.0)	<0.001	3.0	2.1–4.2
**Stilbirths**					
Yes	38(25.7)	12193(7.9)	<0.001	4.0	2.8–5.8
No	110(74.3)	141680(92.1)			
**Mean birth weight(Kg±SD)**	2.6(±0.6)	2.9(±0.7)	<0.001		
**Low birth weight(Kg)**					
Yes (<2.5)	50 (34.2)	27324 (17.8)	<0.001	2.4	1.7–3.4
No (2.5 or above)	96(65.8)	125752(82.2)			
**Maternal outcome**					
Alive	132(88.6)	154518(99.6)			
Died	17(11.4)	689(0.4)	<0.001	28.9	17.3–48.1

* Unless specified otherwise data are displayed as number (%).

Comparing with non-SCD deliveries, SCD had a 2.4 risk of delivering low birth weight babies. The risk of low APGAR score at five minutes was 3 times for SCD as for the non-SCD population. A total of 12588 (8.0%) of all the deliveries never survived the first five minutes of birth including 38 (25.7%) of SCD and 12193(7.9%) of the general population, (p<0.001). The risk of a newborn scoring 0 after 1 minute or after 5 minutes was 4 times in SCD as for non-SCD deliveries.

Of the 149 SCD deliveries, deaths occurred in 17 which make the incidence of maternal death of 1141 per 100,000 deliveries compared to 439 per 100,000 deliveries for non SCD deliveries. Thus the risk of dying was 29 times for SCD as for the non-SCD deliveries (Table 2).

**Figure 3:**
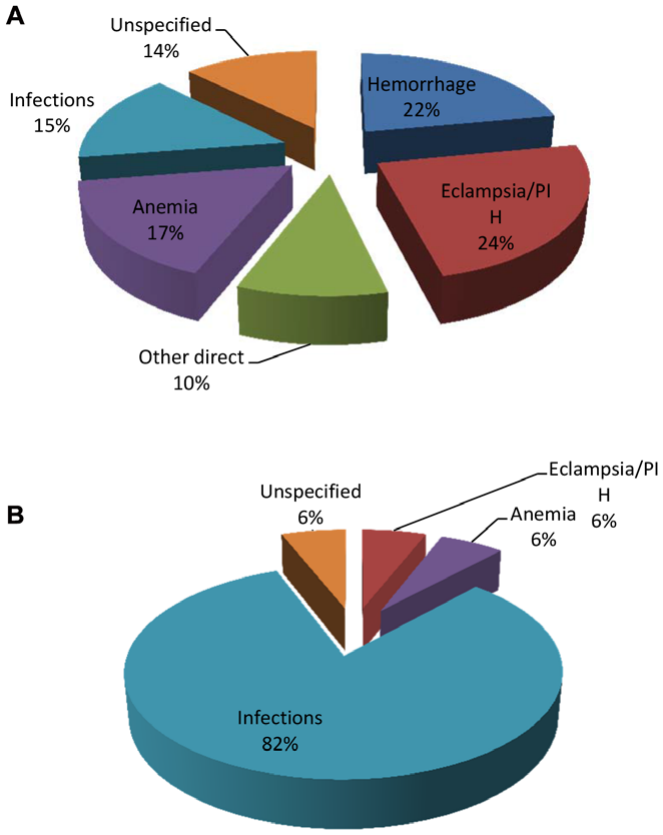
Causes of maternal deaths in non-SCD population(A). (B)Causes of maternal deaths in SCD.

Figure 3A and 3B display the causes of maternal deaths for SCD and non-SCD deliveries. The most frequent causes of deaths among non-SCD were eclampsia/PIH (24%), hemorrhage (22%) and anemia (16%). In contrast, the causes of deaths in 82% of SCD deliveries were infections. These infections include malaria (58.8%), sepsis (11.8%) and HIV/AIDS (11.8%). Hemorrhage was not a major cause of deaths in SCD.

## Discussion

Sickle cell anemia is a major public health problem in Tanzania [11] but more attention has been drawn to SCD in childhood with almost none paid to SCD in pregnancy. As childhood survival continue to improve [20], the burden of SCD in pregnancy will continue to rise as proved by this study. The SCD incidence of 95 per 100, 000 deliveries and a rising trend of SCD deliveries from 76 per 100,000 deliveries in 1999–2002 to over 100 per 100, 0000 deliveries in more recent years, underscores the need for paying more attention to SCD in pregnancy.

The objective of the current study was to compare outcomes of SCD deliveries with those of the general population of women who delivered at MNH without the diagnosis of SCD. We found that SCD deliveries were significantly younger and of lower parity than in non-SCD which is supported by some studies [12] but not all. In the contrary, Serjeant and others have reported older age at delivery in SCD than in non-SCD [21]. It remains unclear if the differences in age at delivery and parity could be attributable by differences in knowledge and practice of timing of pregnancy among SCD women. Although studies show delayed menarche among SCD, they do not generally support early timing of first pregnancy for SCD women [21]–[23]. Timing of conception and spacing of pregnancy is particularly important among SCD and future studies should seek to address this gap in knowledge.

All SCD mothers in the current study sought antenatal care and nearly a third came directly from home to seek delivery at a tertiary hospital. Despite their efforts to seek the best health care services, maternal and perinatal outcomes were generally poor. Compared with non-SCD, SCD deliveries had lower birth weights, poorer APGAR scores at one and five minutes, and increased stillbirth rate. These findings support findings by studies from low income and higher income countries [13], [15]–[16], [18]–[19], [21], [24] indicating common difficulties experienced in improving fetal outcomes even with the standard management measures in place. Unlike some other studies that show increased prematurity rate among SCD [15]–[16], [21], [25], the current study did not find a statistically significant difference in prematurity rate and gestation age at delivery among SCD and non-SCD. This discrepancy could be explained by the failure of this study to take into account of women who did not deliver at MNH, and the presence of multiple factors contributing to prematurity in the study population. A third of all deliveries (31%) took place at the gestation age of less than 37 weeks. Future prospective studies should help to clarify this for Tanzania.

Maternal mortality was very alarming with an incidence of 1149 deaths per 100,000 SCD deliveries compared with 439 per 100,000 deliveries for non SCD in this study and the 1.8% to 9% in West Africa [18]–[19]. In the current study, the risk of death was 29 times higher for SCD as for non-SCD which is much higher than the 7–11 in Jamaica [12]. Nevertheless, this extremely high maternal mortality was comparable with that reported in Nigeria [26]. Excessive maternal deaths can be prevented by improved care as has been the case in developed countries with standard care of SCD in pregnancy where maternal deaths due to SCD is uncommon [16]. With improved prenatal care good outcomes have also been reported in some African settings [18].

In the current study, the most frequent causes of deaths among non-SCD were eclampsia/PIH (24%), hemorrhage (22%) and anemia (16%). In contrast, the causes of deaths in 82% of SCD deliveries were infections with two thirds of them being due to malaria alone. The pathophysiological mechanisms of deaths due to malaria infection in SCD pregnancies are not fully understood. In childhood SCD does not seem to increase the risk of malaria infection although SCD increases the risk of severe morbidity and dying among hospitalized patients [27]–[28]. Whether this is the case for SCD in pregnancy, it remains to be established. What is clear from this study is that interventions to prevent malaria parasitemia in pregnancy, adherence to aseptic techniques during labor and delivery in order to minimize sepsis and HIV screening and treatment should constitute the minimum requirements for care of SCD. Health professionals should be prepared to effectively diagnose and manage SCD in pregnancy. It is also necessary for women with SCD, their partners and families to receive extensive education and counseling on the complications of SCD in pregnancy if these complications are to be minimized. These findings call for urgent need to introduce standard care for SCD women who contemplate pregnancy or become pregnant in Tanzania.

Our study utilized database information which, similar to other retrospective sources, suffered data incompleteness. Incompleteness of some useful socio-demographic variables and intrapartum variables such as induction of labor, premature rupture of membranes and others limited our analysis. Moreover, the diagnosis of SCD was made before admission in all of them and data concerning specific genotypic disorders were lacking. In order to protect validity of the analyzed variables we decided a priori to include variables with not more than 5% missed values and whose distribution of missing values was similar for SCD and non-SCD. This was a hospital based study for women who came for delivery at MNH. It does not contain information on early pregnancy losses. In Dar es Salaam only about a quarter of childhood SCD die in hospital [11], but for SCD in pregnancy this is likely to be different. Statistics for pregnant women in Dar es Salaam [20] indicate a high antenatal care attendance (100%) and hospital delivery (90%) among pregnant women. This implies that our study population may not differ markedly from the community population of delivering women. Strength of this study is that it is one of the largest studies done on SCD in pregnancy in Sub-Saharan Africa and the only one in Tanzania. The findings in this study can be reliably used as baseline for policy change.

## Conclusion

The incidence of SCD delivery at MNH has been on a rise in the past 13 years. The disease is associated with excessive maternal deaths that are by far attributable to infections. Standard guidelines to improve care of SCD in pregnancy are urgently needed in Tanzania.
